# Manifestationen im Zentralnervensystem nach COVID-19

**DOI:** 10.1007/s00115-022-01294-2

**Published:** 2022-05-12

**Authors:** Ameli Gerhard, Harald Prüß, Christiana Franke

**Affiliations:** 1 Klinik für Neurologie und Experimentelle Neurologie, Charité – Universitätsmedizin Berlin, Hindenburgdamm 30, 12200 Berlin, Deutschland; 2 Deutsches Zentrum für Neurodegenerative Erkrankungen (DZNE) Berlin, Berlin, Deutschland

**Keywords:** Enzephalitis, Myelitis, Autoantikörper, Hyperinflammation, Immuntherapie, Encephalitis, Myelitis, Autoantibodies, Hyperinflammation, Immunotherapy

## Abstract

Zahlreiche Erkrankungen des Zentralnervensystems sind insbesondere in der Postakutphase nach einer Infektion mit SARS-CoV‑2 („severe acute respiratory syndrome coronavirus 2“) beschrieben. Diese umfassen neuroimmunologisch vermittelte Erkrankungen wie Enzephalopathien, Enzephalitiden, Myelitiden, ADEM (akute disseminierte Enzephalomyelitis), ANHLE (akute nekrotisierende hämorrhagische Leukoenzephalitis) und NMOSD (Neuromyelitis-optica-Spektrum-Erkrankungen), aber auch andere wie PRES (posteriores reversibles Enzephalopathiesyndrom), OMAS (Opsoklonus-Myoklonus-Ataxie-Syndrom) sowie zerebrovaskuläre Erkrankungen. Ein para- oder postinfektiöser Zusammenhang wird diskutiert, jedoch sind pathophysiologische Mechanismen bislang unbekannt. Ursächlich könnte eine virusgetriggerte Überaktivierung des Immunsystems mit Hyperinflammation und Zytokinsturm, aber möglicherweise auch die Bildung spezifischer Autoantikörper gegen Gewebe des Zentralnervensystems sein. Eine direkte Schädigung durch die Invasion von SARS-CoV‑2 in das Gehirn oder das Rückenmark scheint keine relevante Rolle zu spielen. Eine exakte klinische Phänotypisierung und Einleitung von Zusatzdiagnostik, auch zum Ausschluss anderer Ursachen, ist empfohlen. Bislang existieren noch keine medikamentösen Therapieoptionen zur Behandlung von ZNS-Manifestationen beim Long-COVID(„coronavirus disease“)-Syndrom. Erste Befunde zu Inflammation und Autoimmunität sind jedoch vielversprechend und könnten zu neuen Therapieansätzen führen.

## Abstract (en)

Numerous diseases of the central nervous system (CNS), especially in the postacute phase after an infection with severe acute respiratory syndrome coronavirus 2 (SARS-CoV-2) have been described. These include neuroimmunologically mediated diseases, such as encephalopathy, encephalitis, myelitis, acute disseminated encephalomyelitis (ADEM), acute necrotizing hemorrhagic leukoencephalitis (ANHLE) and neuromyelitis optica spectrum disorder (NMOSD) as well as others, such as posterior reversible encephalopathy syndrome (PRES), opsoclonus myoclonus ataxia (OMA) and cerebrovascular diseases. A parainfectious or postinfectious association is discussed but the pathophysiological mechanisms are so far unknown. Underlying mechanisms could be a virus-triggered overactivation of the immune system with hyperinflammation and cytokine storm but possibly also the development of specific autoantibodies against CNS tissue. Direct damage due to the invasion of SARS-CoV‑2 into the brain or spinal cord does not seem to play a relevant role. An exact clinical phenotyping and initiation of additional diagnostics are recommended, also to rule out other causes. To date no medicinal treatment options for CNS manifestations of long COVID exist; however, first results regarding inflammation and autoimmunity are promising and could lead to new treatment approaches.

## Hintergrund

Das Long-COVID(„coronavirus disease“)-Syndrom (LCS) umfasst vielfältige Symptome, die sämtliche Organsysteme [43] betreffen können. Etwa 10–40 % der an COVID-19 Erkrankten berichten, an residuellen oder neu aufgetretenen Beschwerden zu leiden [64]. Die häufig fehlende Kontrollgruppe in den bislang hierzu publizierten Daten birgt das Risiko der Überschätzung des LCS. Eine französische Querschnittsstudie konnte zeigen, dass auch Menschen, die keine nachgewiesene COVID-19-Erkankung erlitten haben, in ähnlichem Maße von residuellen Symptomen, vereinbar mit denen eines LCS, berichten [47].

Die Terminologie zur Bezeichnung und zeitlichen Abgrenzung des LCS ist im allgemeinen und wissenschaftlichen Sprachgebrauch mitunter uneinheitlich. Nach den britischen, mittlerweile international verwendeten NICE (National Institute for Health and Care Excellence)-Guidelines [52] umfasst das LCS Symptome, die 4 Wochen nach der Akutinfektion fortbestehen. Das Post-COVID-19-Syndrom (PCS) beschreibt Beschwerden, die auch noch 12 Wochen nach der Akutinfektion vorhanden sind. Wir werden im vorliegenden Artikel dieser zeitlichen Definition folgen. Mit Postakutphase ist der Zeitraum bis 4 Wochen nach der Akutinfektion gemeint.

**Figure 1:**
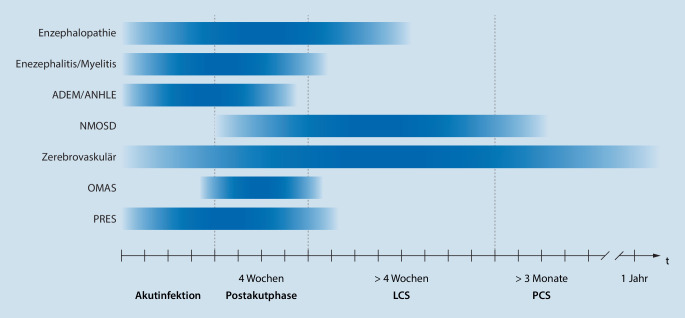

Die häufigsten neurologischen Manifestationen des LCS und PCS sind Fatigue und neurokognitive Störungen [11, 12, 60], die in weiteren Artikeln dieser Ausgabe von *Der Nervenarzt* diskutiert werden. Dieser Beitrag fokussiert sich auf andere berichtete Manifestationen im Zentralnervensystem (ZNS) des LCS und PCS.

## Syndrome und Symptome des ZNS nach COVID-19

Syndrome des ZNS umfassen Enzephalopathien, entzündliche Erkrankungen wie Enzephalitis und Myelitis, zerebrovaskuläre Erkrankungen und andere, bislang unvollständig verstandene Syndrome. Die Symptome des ZNS reichen von diffusen Beschwerden wie Bewusstseinsstörungen und Kopfschmerzen zu (zentralen) fokalneurologischen Defiziten wie Paresen, Ataxie und Sehstörungen (Abb. 1).

### Enzephalopathie

Als Enzephalopathie wird eine diffuse Funktionsstörung des Gehirns mit qualitativer und quantitativer Bewusstseinsstörung, aber auch fokalneurologischen Defiziten und/oder epileptischen Anfällen beschrieben. Sie ist als neuropsychiatrische Komplikation der akuten COVID-19-Erkrankung mittlerweile bekannt [32]. Es ist anzunehmen, dass die Ätiologie einer Enzephalopathie bei akut und schwer erkrankten COVID-19-Patient:innen multifaktoriell ist.

Parainfektiöse Enzephalopathien traten im Mittel 13 Tage nach Beginn der Akutsymptomatik auf [71]. Dennoch gibt es auch einige Berichte über persistierende oder verspätet auftretende Enzephalopathien im Rahmen einer leichten COVID-19-Erkrankung: Jozuka und Kolleg:innen berichten z. B. über eine 55-jährige, zuvor gesunde Frau, die 15 Tage nach einer milden COVID-19-Erkrankung eine schwere und langanhaltende Enzephalopathie entwickelte. Im Liquor fanden sich erhöhte Zytokinwerte. Nach einer Kortisonstoßtherapie besserten sich die klinischen Symptome und die Zytokinwerte sanken [36]. Aus den USA wird von drei Patient:innen mit intensiv- und beatmungspflichtigem COVID-19-Verlauf berichtet, die eine schwere prolongierte Enzephalopathie zeigten. Bei allen drei führte ein probatorischer Therapieversuch mit 5 Zyklen Plasmapherese zu einer signifikanten Verbesserung der Klinik und Normalisierung eines zuvor pathologischen Elektroenzephalogramms [66]. Eigene Untersuchungen von Patient:innen mit prolongierter Enzephalopathie und intensivmedizinischem COVID-19-Verlauf zeigten in 9 von 12 Fällen eine Besserung des klinischen Zustandes unter Behandlung mit intravenösen Immunglobulinen (IVIGs) während der Postakutphase [31]. Diese Arbeiten stützen die Annahme, dass der COVID-19-Enzephalopathie ein immunvermittelter Mechanismus zugrunde liegt.

### Enzephalitis und Myelitis

Im Unterschied zur Enzephalopathie findet sich bei der Enzephalitis ein entzündlich veränderter Liquor. Ätiologisch unterscheidet man direkt erregerbedingte und autoimmunvermittelte Enzephalitiden. Bei letzteren gibt es sowohl paraneoplastische als auch parainfektiöse, kreuzreaktive Autoantikörper (Auto-Ak), wobei hier die NMDAR(N-Methyl-D-Aspartat-Rezeptor)-Enzephalitis nach HSV(Herpes-simplex-Viren)-Infektion das bekannteste Beispiel ist [62]. Auch hier liegen bereits multiple Fallberichte vor, die über eine Enzephalitis oder Enzephalomyelitis als mögliche Komplikation der akuten SARS-CoV-2(„severe acute respiratory syndrome coronavirus 2“)-Infektion berichten. Als antineuronale Autoantikörper (Ak), die im Rahmen einer akuten COVID-19-Erkrankung auftreten, konnten NMDAR-, CASPR2(Contactin-assoziiertes Protein-2)-, MOG(Myelin-Oligodendrozyten-Glykoprotein)-, GAD(Glutamatdecarboxylase)- und GD1a(Gangliosid)-Ak identifiziert werden [55].

Da bei den parainfektiösen Enzephalitiden eine para- bzw. postinfektiöse Autoimmunreaktion angenommen wird, ist es nicht verwunderlich, dass diese auch in der Postakutphase von COVID-19 auftreten. Die erkrankten Patient:innen waren zwischen 20 und 81 Jahre alt, und die Symptomatik trat 2 bis 4 Wochen nach der Akutinfektion auf, meistens im Anschluss an einen mildem Akutverlauf. In den meisten Fällen konnten keine etablierten antineuronalen Ak nachgewiesen werden [14, 22, 25, 26, 50, 56, 58, 68]. In drei Fällen wurden jedoch GAD- (im Serum, bei klinisch passender Zerebellitis und Rhombenzephalitis; [17, 33]) sowie GAD- in Kombination mit NMDAR-Ak (im Liquor, bei limbischer Enzephalitis; [75]) nachgewiesen. Hervorzuheben sind auch zwei Fälle, in denen es während der Enzephalitis zu einem ausgeprägten Parkinsonismus kam und im Fluordesoxyglukose-Positronenemissionstomographie (FDG-PET) ein kortikaler Hypometabolismus festgestellt wurde [50]. Dies führt zu der Mutmaßung, dass die Post-COVID-19-Enzephalitis – ähnlich der Encephalitis lethargica nach der Spanischen Grippe – bei einer kleinen Subgruppe von Patient:innen zu postakut auftretenden Bewegungsstörungen als Komplikation einer COVID-19-Erkrankung führen kann [7, 28, 42].

Auch die transverse Myelitis kann durch virale Infektionen ausgelöst werden [38] und wurde ebenfalls in der Postakutphase von COVID-19 beschrieben [2], in einem Fall mit begleitender akuter motorischer axonaler Neuropathie (AMAN) und dem Nachweis von GD1b-Ak im Serum [45].

### ADEM und ANHLE

Akute disseminierte Enzephalomyelitis (ADEM) und die Variante akute nekrotisierende hämorrhagische Leukoenzephalitis (ANHLE) wurden seit Beginn der Pandemie im Zusammenhang mit COVID-19 beschrieben. ADEM ist eine demyelinisierende, autoimmun vermittelte Erkrankung, die meist parainfektiös und in seltenen Fällen postvakzinal auftritt. Sie betrifft vor allem Kinder und Jugendliche, kann aber auch bei Erwachsenen vorkommen [59].

Alle ANHLE-Patient:innen hatten einen symptomatischen COVID-19-Akutverlauf

Es gibt mehrere Fallberichte über das Auftreten von ADEM und ANHLE in der Postakutphase von COVID-19-Erkrankungen, sowohl bei Erwachsenen als auch bei Kindern. Gelibter und Kolleg:innen haben die adulten Fallbeispiele kürzlich zusammengetragen [23]: Insgesamt gab es 20 Patient:innen mit ADEM und 23 mit ANHLE. Das Alter der Erkrankten lag zwischen 40 und 53 Jahren. Die Erkrankung trat im Zeitraum von 9 bis 20 Tagen nach der Akutinfektion und damit in der postinfektiösen Phase auf. Im Liquor wurde SARS-CoV‑2 in einem Fall, MOG- und AQP4(Aquaporin-4)-Ak in keinem Fall nachgewiesen. Patient:innen mit ADEM hatten einen milden oder asymptomatischen COVID-19-Akutverlauf, wohingegen alle ANHLE-Patient:innen einen symptomatischen und 50 % von ihnen einen schweren Verlauf mit intensivstationärem Aufenthalt erlitten hatten. Therapie und Outcome waren sehr unterschiedlich, insgesamt war das Outcome bei ANHLE-Patient:innen aber schlechter. Bei ADEM-Patient:innen konnte das Fehlen einer Enzephalopathie als positiver prognostischer Faktor identifiziert werden.

Die Prävalenz der gesichert im Zusammenhang mit COVID-19 aufgetretenen Fälle von ADEM und ANHLE ist insgesamt niedriger als vor der Pandemie [15, 23] und es konnten epidemiologische Unterschiede zwischen prä- und postpandemischen Fällen festgestellt werden [23, 44]. SARS-CoV‑2 kann allerdings, ähnlich wie andere Viren, als infektiöser Trigger für ADEM und ANHLE fungieren und in der Postakutphase diese Erkrankungen als potenzielle Komplikationen auslösen.

### Andere demyelinisierende Erkrankungen

Daten zum Auftreten einer Multiplen Sklerose im Anschluss an eine COVID-19-Erkrankung liegen aktuell nicht vor. SARS-CoV‑2 könnte hier jedoch als viraler Trigger [58] ähnlich einer bei der Multiplen Sklerose pathophysiologisch diskutierten Epstein-Barr-Virus(EBV)-Reaktivierung bzw. EBV-Infektion fungieren [10].

Weiterhin gibt es Fallberichte über das Auftreten AQP4-seropositiver Neuromyelitis-optica-Spektrum-Erkrankungen (NMOSD) nach COVID-19 [24, 34], in einem Fall mit einer vergleichsweise langen Latenz von 3 Monaten nach der Akutinfektion.

### Vaskuläre Erkrankungen

Metaanalysen zeigen einen Zusammenhang der akuten COVID-19-Erkrankung mit dem Auftreten zerebrovaskulärer Ereignisse, insbesondere ischämischer Schlaganfälle v. a. bei kritisch kranken Patient:innen und solchen mit vaskulären Risikofaktoren. Als Pathomechanismus scheint hier maßgeblich ein Zusammenspiel aus einem hyperinflammatorischem und hyperkoagulativem Zustand, getriggert durch die Virusinfektion, vorzuliegen [51, 74]. Intrazerebrale Blutungen treten sowohl als Komplikation intensivstationärer Therapieeskalation (z. B. bei therapeutischer Antikoagulation oder extrakorporaler Membranoxygenierung (ECMO)) als auch im Rahmen der Akutinfektion selbst auf [40].

Es besteht ein substanziell erhöhtes Risiko für Schlaganfälle und TIA

Gleiches gilt für das Auftreten zerebrovaskulärer Ereignisse mit längerem Abstand zur COVID-19-Erkrankung: In einer großen Kohortenanalyse konnte gezeigt werden, dass bis zu einem Jahr nach einer COVID-19-Erkrankung ein substanziell erhöhtes Risiko für Schlaganfälle und transitorische ischämische Attacken (TIA) besteht – unabhängig von Alter und kardiovaskulären Risikofaktoren der Patient:innen. Ein schwerer Akutverlauf war auch hier mit einem höheren Risiko verbunden [77].

In zwei Fallberichten über das Auftreten ischämischer Schlaganfälle als Manifestation eines LCS/PCS bei vergleichsweise jungen Patient:innen ohne Vorerkrankungen fanden sich erhöhte D‑Dimere [6, 57]. Bei einer weiteren Patientin manifestierte sich ein CADASIL(„cerebral autosomal dominant arteriopathy with subcortical infarcts and leukoencephalopathy“)-Syndrom als potenzielle postinfektiöse Komplikation [65].

Zerebrale Sinus- und Venenthrombosen (SVT) sind eine seltene Komplikation von COVID-19 [5]. In 2 Fällen wurde über das Auftreten einer SVT in der Postakutphase berichtet (4 Tage bis 2 Wochen nach der Akutinfektion; [1, 67]).

### Andere ZNS-Syndrome

#### Opsoklonus-Myoklonus-Ataxie-Syndrom

Das Opsoklonus-Myoklonus-Ataxie-Syndrom (OMAS) ist eine bislang unvollständig verstandene Erkrankung, welche sich durch das plötzliche Auftreten der namensgebenden Symptome auszeichnet, wobei nicht alle drei Symptome gleichzeitig vorliegen müssen. Es wird bei Erwachsenen meist durch paraneoplastische antineuronale Ak ausgelöst oder durch onkoneuronale Ak angezeigt [53]. Parainfektiöse Ursachen sind weniger häufig, wurden aber auch bereits im Zusammenhang mit anderen Viruserkrankungen berichtet [39].

Im Zusammenhang mit COVID-19 gibt es mehrere Fallberichte über das Auftreten eines OMAS in der Postakutphase – die meisten Patient:innen entwickelten die Symptome etwa 2 Wochen (10 Tage bis 6 Wochen) nach der Akutinfektion [63]. Lediglich bei zwei Fällen trat ein OMAS in der Akutphase von COVID-19 auf [16]. Die meisten Patienten waren männlich, das Alter lag zwischen 39 und 83 Jahren. Blut- und Liquoruntersuchungen, inkl. onkoneuronaler Ak, waren in allen Fallberichten unauffällig. In den Fällen, in welchen eine kontrastmittelgestützte Magnetresonanztomographie durchgeführt wurde, konnten keine Auffälligkeiten festgestellt werden. In einem Fall wurden GFAP(„glial fibrillary acidic protein“)-Ak im Serum gefunden, welche 3 Monate nach der Erkrankung nicht mehr nachweisbar waren [4]. Die meisten Patient:innen erhielten immunsuppressive Therapien (hoch dosiertes Methylprednisolon, IVIGs und/oder Plasmapherese), teilweise in Kombination mit antikonvulsiver Medikation und erholten sich hierunter vollständig.

#### PRES

Das posteriore reversible Enzephalopathiesyndrom (PRES) ist ein pathophysiologisch noch unvollständig verstandenes klinisch-radiologisches Syndrom, das durch hypertensive Krisen oder zytotoxische Substanzen wie Zytostatika ausgelöst werden kann [3]. Als akute neurologische Manifestation von COVID-19 wurde es bereits beschrieben [37, 61].

Wir konnten zwei Fallberichte identifizieren, in denen ein PRES nach bereits überstandener schwerer COVID-19-Erkrankung auftrat. Zum einen wird von einer 61-jährigen Frau mit intensivpflichtigem Akutverlauf [49], zum anderen von einer 90-jährigen Frau mit nichtintensivpflichtiger COVID-19-Pneumonie berichtet [41]. Die Symptomatik entwickelte sich auch hier jeweils in der Postakutphase (3 bis 5 Wochen nach Beginn der Akuterkrankung). In beiden Fällen konnte unter antikonvulsiver Therapie eine klinische und bildgebende Besserung erreicht werden.

## Pathomechanismen bei LCS und PCS

Verschiedene Pathomechanismen bzw. ihr Zusammenspiel werden als Ursache des LCS und PCS diskutiert. Hierzu zählen insbesondere para- und postinfektiöse Autoimmunmechanismen, hyperinflammatorische Prozesse, Koagulopathien und zerebrale Mikrozirkulationsstörungen [35]. Eine direkte ZNS-Schädigung durch das Virus selbst wird mittlerweile für nachrangig gehalten. Dafür spricht auch, dass nur in seltenen Fällen bei schwer und akut an COVID-19 erkrankten Patient:innen mit neurologischen Manifestationen SARS-CoV‑2 mittels PCR („polymerase chain reaction“) im Liquor festgestellt werden konnte [27]. Eine fehlende intrathekale Anti-SARS-CoV-2-Ak-Synthese spricht zudem gegen eine persistierende ZNS-Infektion als Ursache für neuropsychiatrische Symptome im Rahmen des LCS/PCS [70].

Im Hirnstamm zeigt sich ein besonders hohes Auftreten von Mikrogliaknötchen

In neuropathologischen Untersuchungen zeigen sich inflammatorische Veränderungen des Hirnstamms [46], aber auch im Bulbus olfactorius und entlang des Riechtraktes [48]. Dies wird als mögliche Eintrittspforte in das ZNS diskutiert. Allerdings bestehen bezüglich des Auftretens von Mikrogliakativierung Unterschiede zwischen Hirnstamm und Bulbus olfactorius. So zeigt sich im Hirnstamm ein besonders hohes Auftreten von Mikrogliaknötchen, hinweisend auf eine Neuroinflammation bedingt durch Einwanderung von T‑Killerzellen und anderen Immunzellen [69].

### Para- und postinfektiöse Autoantikörperbildung

Das Phänomen der para- oder postinfektiösen virusgetriggerten Auto-Ak-Bildung ist von anderen Viruserkrankungen wie der HSV-Enzephalitis bekannt [62]. Die Kreuzreaktivität antiviraler Ak mit körpereigenen Oberflächenstrukturen ist auch für andere neurologische Erkrankungen wie das Guillain-Barré-Syndrom bereits beschrieben [73]. Für einen solchen Mechanismus sprechen insbesondere der Zeitpunkt des Auftretens von Enzephalitiden, Myelitiden, ADEM und OMAS in der Postakutphase von COVID-19 sowie das gute Ansprechen auf immunsuppressive Therapie (Methylprednisolon, IVIGs oder Plasmapherese).

Dass Autoreaktivität bei COVID-19 eine wichtige Rolle spielt, konnte bereits mehrfach gezeigt werden [72]. Der häufig fehlende Nachweis bekannter, oft syndromspezifischer antineuronaler Ak in Liquor oder Serum macht diesen Mechanismus nicht unwahrscheinlicher, da etliche antineuronale Ak eine Rolle spielen dürften und noch nicht bekannt sind. Bei intensivpflichtigen Patient:innen mit neurologischen Manifestationen konnte während der Akutinfektion eine starke Ak-Bindung gegen neuronale Strukturen gezeigt werden [20]. Dies scheint zumindest für eine Subgruppe der Patient:innen mit primär kognitiven Defiziten im Rahmen eines PCS gleichermaßen zu gelten [19].

Die Elimination von Auto-Ak gegen G‑Protein-gekoppelte Rezeptoren wird als als therapeutische Option evaluiert

Eine genaue Zuordnung von klinischer Symptomatik zu einem Autoantikörperbefund bzw. zu einer spezifischen Hirnregion steht bislang aus. Die Elimination funktioneller Auto-Ak gegen G‑Protein-gekoppelte Rezeptoren [76] wird aktuell auch als therapeutische Option weiter untersucht, nachdem in einem Fall [30] eine positive Wirkung auf das PCS gezeigt werden konnte. Insgesamt erhielten bis heute drei Patient:innen (zwei Männer und eine Frau zwischen 39 und 51 Jahren, davon ein Fall publiziert) mit schweren neurologischen PCS-Symptomen (u. a. starke Fatigue, kognitive Einschränkungen, Koordinationsschwierigkeiten, Paresen) in individuellen Heilversuchen das ursprünglich gegen Herzinsuffizienz entwickelte Medikament BC 007 [21]. Im publizierten Fall zeigte sich über einen Beobachtungszeitraum von 4 Wochen eine deutliche Verbesserung der klinischen Symptome sowie eine anhaltende Neutralisierung der Auto-Ak gegen G‑Protein-gekoppelte Rezeptoren [30].

### Hyperinflammation und Zytokinsturm

Erhöhte Serumspiegel proinflammatorischer Zytokine sind mit einem schlechteren Outcome der akuten COVID-19-Erkrankung assoziiert [13], weshalb postuliert wurde, dass (neurologische) Komplikationen und schwere Verläufe durch hyperinflammatorische Prozesse wie den sog. Zytokinsturm bedingt sein könnten. Zytokine, v. a. Interleukin‑6, erhöhen die Permeabilität der Blut-Hirn-Schranke, wodurch es zur Aktivierung von Mikroglia und daraus folgenden weiteren Schädigungen des ZNS kommt. Eine zytokin- oder noxenvermittelte Störung der Blut-Hirn-Schranke mit nachfolgender endothelialer Dysfunktion bzw. Astrozytenschädigung wird auch für das PRES und die NMOSD als potenzieller Pathomechanismus diskutiert [3, 24].

### Gerinnungsstörung und Mikrozirkulationsstörungen

Das vermehrte Auftreten zerebrovaskulärer Ereignisse in der Postakutphase von COVID-19 lässt Gerinnungsstörungen als pathophysiologischen Mechanismus vermuten. Hierfür spricht, dass sich während der Akutinfektion häufig erhöhte D‑Dimer-Werte finden. Zudem wurden Cardiolipin-Ak (Lupus-Antikoagulans), die in der Akutinfektion als Marker für die Schwere der Erkrankung gelten, teilweise auch bei PCS-Patient:innen festgestellt [9]. Auch eine zytokinvermittelte zerebrale Hypoperfusion ist für bestimmte ZNS-Manifestationen des LCS/PCS denkbar. Die Entfernung G‑Protein-gekoppelter Rezeptor-Ak soll unter anderem zu einer verbesserten retinalen Durchblutung führen und somit eine möglicherweise bestehende endotheliale Dysfunktion verbessern [29].

## Zusammenfassung und Ausblick

Die ZNS-Manifestationen des LCS und PCS sind vielfältig. Insbesondere während der Postakutphase sind entzündliche, autoimmune, demyelinisierende und vaskuläre Syndrome beschrieben, die den ZNS-Manifestationen der Akutinfektion ähnlich sind. Die Aufklärung der Krankheitsmechanismen stehen beim LCS und PCS erst am Anfang und es sind weitere umfassende klinische und grundlagenwissenschaftliche Arbeiten zum besseren Verständnis notwendig.

Ein kausaler Zusammenhang der ZNS-Symptome mit der SARS-CoV-2-Infektion ist auch in der Akutphase nicht immer eindeutig. Dies gilt gleichermaßen bzw. noch mehr für das Auftreten von Symptomen in der Postakutphase und im weiteren zeitlichen Verlauf. Unserer Erfahrung nach sind prämorbider Status und Verlauf der Akutinfektion nur bedingt hilfreich im Hinblick darauf, ob und welche Beschwerden residuell nach COVID-19 bestehen. Beschrieben ist das gehäufte Auftreten von Kopfschmerzen und Fatigue bei Patient:innen, die auch schon während der Akutinfektion unter Kopfschmerzen litten [18]. Spezifische Biomarker für LCS/PCS existieren bislang nicht. Umso wichtiger ist die Einleitung einer umfassenden (Differenzial‑)Diagnostik entsprechend den aktuellen DGN-Leitlinien [78], um potenziell anders zu behandelnde Ursachen auszuschließen bzw. adäquat zu behandeln. Insbesondere bei Patient:innen mit intensivpflichtigem COVID-19-Akutverlauf und anhaltenden neurologischen Beschwerden ist eine ursächliche Zuordnung der neurologischen Manifestationen besonders herausfordernd, da z. B. residuelle kognitive Störungen auch im Rahmen eines PICS („post intensive care syndrome“) bestehen können [54].

Es existieren derzeit keine kausalen oder etablierten Therapien zur Behandlung des LCS/PCS [8]. Erste Befunde zu Inflammation und Autoimmunität sind vielversprechend und könnten zu neuen Therapieansätzen führen. Berichte über eingeleitete medikamentöse Behandlungen sind bislang allerdings auf individuelle Heilversuche in kleinen Patientengruppen limitiert, sodass größere, placebokontrollierte Studien notwendig sind, um potenzielle Behandlungsstrategien zu untersuchen.

## Fazit für die Praxis

- Nach der Akutinfektion mit SARS-CoV‑2 („severe acute respiratory syndrome coronavirus 2“) können in Abhängigkeit vom zeitlichen Abstand residuelle oder neu aufgetretene Symptome bestehen. Unterschieden werden die Postakutphase (Zeitraum bis 4 Wochen nach der Akutinfektion), das Long-COVID(„coronavirus disease“)-19-Syndrom (ab 4 Wochen nach der Akutinfektion) und das Post-COVID-19-Syndrom (ab 12 Wochen nach der Akutinfektion).
- Neuroimmunologische Krankheitsbilder des Zentralnervensystems (ZNS) nach COVID-19 umfassen Enzephalitiden, Enzephalopathien, ADEM (akute disseminierte Enzephalomyelitis) und Myelitiden. Es besteht ein erhöhtes Risiko für zerebrovaskuläre Erkrankungen im ersten Jahr nach der Akutinfektion.
- An pathophysiologischen Mechanismen werden para- und postinfektiöse Autoimmunmechanismen, hyperinflammatorische Prozesse, Koagulopathien und zerebrale Mikrozirkulationsstörungen diskutiert. Ein direkter Schaden durch das Virus selbst oder Antikörper gegen das Virus im ZNS erscheinen nachrangig.
- Autoreaktivität spielt bei Symptomen im Nachgang an COVID-19 eine wesentliche Rolle.

## Abkürzungen

ADEM: Akute disseminierte Enzephalomyelitis
Ak: Antikörper
AMAN: Akuter motorische axonale Neuropathie
ANHLE: Akute nekrotisierende hämorrhagische Leukoenzephalitis
Auto-Ak: Autoantikörper
EBV: Epstein-Barr-Virus
IVIGs: Intravenöse Immunglobuline
LCS: Long-COVID-Syndrom
NICE: National Institute for Health Care and Excellence
NMOSD: Neuromyelitis-optica-Spektrum-Erkrankungen
OMAS: Opsoklonus-Myoklonus-Ataxie-Syndrom
PCS: Post-COVID-19-Syndrom
PICS: Post intensive care syndrome
PRES: Posteriores reversibles Enzephalopathiesyndrom
SARS-CoV‑2: Severe acute respiratory syndrome coronavirus 2
SVT: Zerebrale Sinus- und Venenthrombosen
TIA: Transitorische ischämische Attacke
ZNS: Zentrales Nervensystem
